# The Environment of Regulatory T Cell Biology: Cytokines, Metabolites, and the Microbiome

**DOI:** 10.3389/fimmu.2015.00061

**Published:** 2015-02-18

**Authors:** Romy E. Hoeppli, Dan Wu, Laura Cook, Megan K. Levings

**Affiliations:** ^1^Department of Surgery, Child and Family Research Institute, University of British Columbia, Vancouver, BC, Canada

**Keywords:** regulatory T cells, FOXP3, cytokines, metabolites, microbiome, plasticity, environment, immune regulation

## Abstract

Regulatory T cells (Tregs) are suppressive T cells that have an essential role in maintaining the balance between immune activation and tolerance. Their development, either in the thymus, periphery, or experimentally *in vitro*, and stability and function all depend on the right mix of environmental stimuli. This review focuses on the effects of cytokines, metabolites, and the microbiome on both human and mouse Treg biology. The role of cytokines secreted by innate and adaptive immune cells in directing Treg development and shaping their function is well established. New and emerging data suggest that metabolites, such as retinoic acid, and microbial products, such as short-chain fatty acids, also have a critical role in guiding the functional specialization of Tregs. Overall, the complex interaction between distinct environmental stimuli results in unique, and in some cases tissue-specific, tolerogenic environments. Understanding the conditions that favor Treg induction, accumulation, and function is critical to defining the pathophysiology of many immune-mediated diseases and to developing new therapeutic interventions.

## Introduction

Regulatory T cells (Tregs) are a suppressive subset of CD4^+^ T helper (Th) cells important for the regulation of immune responses. The best-characterized Tregs are defined by expression of the transcription factor forkhead box protein 3 (FOXP3) and demethylation of the Treg-specific demethylated region (TSDR) in the FOXP3 locus. Demethylation of this element is thought to be crucial to maintain the stable, high expression of FOXP3 necessary for lineage stability and suppressive function (1, 2). Additional Treg markers include constitutive expression of the high-affinity IL-2Rα chain (CD25) and cytotoxic T lymphocyte-associated antigen 4 (CTLA-4) (3), along with low expression of the IL-7Rα chain (CD127) (4, 5). CD4^+^CD25^+^FOXP3^+^ Tregs can be divided into two main types: thymically derived Tregs (tTregs) and peripherally derived Tregs (pTregs) (6). Although it is difficult to distinguish between tTregs and pTregs phenotypically, both are thought to have an essential role in immune regulation (7).

Because of their immunoregulatory function, Tregs are an attractive therapeutic target in many different immune-mediated diseases, including transplantation, autoimmunity, and autoinflammation (8). An emerging concept is that Tregs are functionally specialized to their local environments (9), with the local milieu of cytokines, metabolites, and catabolites having major effects on the phenotype and function of these cells. In this review, we discuss current knowledge on how environmental factors affect Treg development, maintenance, and function, focusing on key recent findings in the area of cytokines, metabolites, and the microbiome.

## Cytokines

### The role of cytokines in tTreg development in the thymus

Development of tTregs in the thymus is critically dependent on signals from the T cell receptor (TCR), CD28, and cytokines. Of particular importance are cytokines that signal via the common γ chain (γ_c_), (10, 11), a topic that has been extensively reviewed (12, 13) (Table 1). Although most data suggest that IL-2 provides the essential signal to CD25^+^FOXP3^−^ single positive tTreg precursors to differentiate into FOXP3^+^ cells, in the absence of IL-2, IL-15 provides a compensatory mechanism (14). In addition, a recent report found that CD25^−^FOXP3^+^ precursors have a specific requirement for IL-15 signaling to develop into tTregs *in vitro* and *in vivo* (15). Notably, while a complete absence of signaling from γ_c_ cytokines leads to a total lack of Tregs (16), in IL-2/IL-15-deficient mice a few Tregs remain (17). These findings suggest there other cytokines that signal through γ_c_ that can partially substitute for IL-2 and IL-15 in instructing tTreg development. An unanswered question is what cells in the thymus make IL-2, and/or other γ_c_ cytokines, and under what conditions? As dendritic cells (DCs) have been shown to make IL-2 (18) and are present in the human thymic medulla in close proximity to developing tTregs (19) they are an obvious candidate, but this has yet to be experimentally investigated.

**Table 1:** **Summary of cytokines that influence Tregs**.

Treg stage	Cytokine	Species	Cytokine function	Reference
Thymic development	IL-2,IL-15	Mouse	Drives development of tTregs by inducing FOXP3 via STAT5	(14, 16, 210)
	IL-7	Mouse	Promotes development of Tregs in absence of IL-2/IL-15	(17)
	TGF-β	Mouse	Induces FOXP3 expression	(20, 211)
Peripheral development	IL-2	Mouse	Critical for TGF-β-induced pTreg development	(23, 212)
			Decreases IL-6R expression, prevents Th17 differentiation	(213)
	TGF-β	Mouse	Induces FOXP3 expression in naïve CD4^+^ T cells *in vitro* and *in vivo*	(24, 214)
		Human	Induces FOXP3 expression in naïve CD4^+^ T cells *in vitro*	(31, 215, 216)
	TNF-α	Mouse	Impairs TGF-β-induced differentiation of pTregs	(92)
Homeostasis	IL-2	Mouse	Up-regulates pro-survival proteins	(51, 52)
			Involved in pTreg homeostasis	(14)
			Maintains Treg GATA3 expression, which suppresses T-bet and RORγt induction	(54–56)
		Mouse/Human	Controls size of Treg pool *in vivo*	(53)
			Induces and stabilizes FOXP3, regulates key Treg-signature molecules	(217–219)
	IL-7	Mouse	Promotes homeostasis of IL-7Rα^+^ memory Tregs in the skin	(57)
	IL-15	Mouse	Promotes homeostasis of IL-15Rβ^+^ memory Tregs accumulating with age	(58)
	IL-33	Mouse	Induces proliferation of colonic ST2^+^ Tregs, increases TGF-β-induced differentiation of ST2^+^ Tregs *in vitro*	(84, 85)
			Induces CD4^+^ FOXP3^+^ Treg proliferation *in vivo*	(81–83)
Function	TNF-α	Human	Reduces FOXP3 mRNA and protein expression levels in Tregs	(89, 90)
			TNF-α-membrane bound: reduces suppressive capacity of Tregs	(91)
		Mouse	Impairs Treg function	(89, 92, 220)
			Augments Treg function and proliferation	(93, 97)
Differentiation	IL-4	Mouse	Induces Th9 differentiation in presence of TGF-β	(221, 222)
	TGF-β + IL-1β, IL-6, IL-21, IL-23, TNF-α	Mouse	Induces Th17 differentiation and maintains Th17 cells	(220, 223–225)
	TGF-β + IL-1β, IL-6, IL-21, IL-23	Human	Induces Th17 differentiation and IL-17 secretion	(225–228)
	IL-23	Mouse	Inhibits Treg differentiation *in vitro* and Treg accumulation in gut	(86, 87)
Th-like Tregs/ex-Tregs	IL-6	Mouse	Induces IL-17 secretion and conversion of Tregs to Th17	(101)
	IL-1β, IL-2, IL-6, IL-15, IL-21, IL-23	Human	Combinations of these cytokines induce IL-17 secretion by Tregs	(102, 103, 105, 107, 112)
	IL-12	Human	Induces expression of T-bet, CXCR3, and IFN-γ production in Tregs	(114–116)
	IL-12, IL-27, IFNγ	Mouse	Induces expression of T-bet, CXCR3, and IFN-γ production in Tregs	(106, 109, 110)

In addition to γ_c_ cytokines, TGF-β also has a critical role in tTreg development. In mice, thymocyte apoptosis leads to production of TGF-β by thymic macrophages, DCs, and epithelial cells, leading to TGF-β-induced FOXP3 expression and tTreg differentiation (20). Interestingly, in mice, this apoptosis in the thymus only occurs after birth, providing an explanation for the long-standing finding that murine tTregs only begin to develop 3 days after birth (21). How this finding relates to tTreg development in humans is unknown, but neonatal humans clearly have tTregs (22), so presumably this process occurs long before birth. Although the relative importance of IL-2 versus TGF-β in tTreg differentiation versus survival is a subject of much debate, both of these cytokines are clearly important for this lineage and understanding the biology of this system in humans will be key to developing therapies to boost tTreg development *in vivo*.

### The role of cytokines in pTreg development in the periphery

The appropriate cytokine milieu is also a critical factor for the development of pTregs. In mice, both TGF-β and IL-2 are required to drive the conversion of CD4^+^CD25^−^FOXP3^−^naïve T cells into CD4^+^CD25^+^FOXP3^+^ pTregs (11, 23–25). However, the final outcome of TGF-β signaling is highly influenced by other surrounding cytokines. For example, anti-inflammatory conditions augment the effects of TGF-β, potentiating pTreg development (26). Conversely, pro-inflammatory cytokines (IL-1β, IL-6, IL-21, IL-23, and/or TNF-α) counteract TGF-β-induced FOXP3 expression and instead drive Th17 cell development by enhancing expression of retinoid-related orphan receptor γt (RORγt), the master Th17 lineage transcription factor (27).

Because of their potential application as a cell-based therapy, many groups have explored the cytokine combinations that can drive the differentiation of FOXP3^+^ Tregs *in vitro* (iTregs) from naïve human CD4^+^ T cells. Early evidence suggested that, as for mice, TCR stimulation in the presence of both TGF-β and IL-2 induced FOXP3 expression. However, the interpretation of these data became difficult when it was recognized that all activated human T cells transiently express FOXP3. Indeed, although TGF-β and IL-2-stimulated human CD4^+^ T cells express FOXP3, their TSDR remains methylated (2, 28, 29), a phenotype indicative of cells that are not stably committed to the Treg lineage. In addition, there are controversial findings on whether the resulting cells are suppressive, with some studies finding suppressive function (30), and others not (2, 28, 29, 31). It is important to note that human Treg suppression assays are particularly difficult to interpret when *in vitro* cultured cells are used due to non-specific effects mediated by media consumption and cell killing (32). Therefore, analysis of the TSDR status, and not functional assays, may be a more reliable way to measure human iTreg development. Collectively, these data suggest that while TGF-β may be necessary for differentiation of mouse and human pTregs *in vivo*, it is likely not sufficient, with other unknown environmental factors needed for their full development.

Interestingly, activated human Tregs express high levels of latent TGF-β coupled to latency-associated peptide and bound to the cell surface protein GARP (33–35). Therefore, Tregs themselves can drive the generation of new pTregs by providing a source of TGF-β (36, 37), offering a molecular explanation for a process termed “infectious tolerance” that has been observed for many years in animal models of transplantation (38–40). Mucosal DCs are also a rich source of TGF-β because they express integrin α_v_β_8_, which converts extracellular latent TGF-β to its active form (41, 42). These cells may therefore be particularly important for the differentiation of intestinal pTregs that, as discussed in more detail below, are required for intestinal homeostasis.

Based on evidence that in humans TGF-β alone does not induce robust differentiation of stable Tregs, many studies have sought to define whether addition of other cytokines and/or compounds can enhance the effect (43). The most convincing evidence comes from addition of either the vitamin A metabolite all trans retinoic acid (ATRA, discussed further in the Section “Metabolites” below) or the mTOR inhibitor rapamycin. In mice, ATRA can be effectively generated by mucosal DCs and functions to enhance TGF-β-mediated pTreg generation, (44–46). In humans, suppressive iTregs can be generated with ATRA and TGF-β, but their stability based on the methylation of the TSDR is unknown (47–49). Similarly, addition of rapamycin enhances TGF-β-induced FOXP3 expression (49), and although the stability of these cells is unknown, there is an ongoing clinical trial to test their potential as a cellular therapy in hematopoietic stem cell transplantation (NCT01634217). Notably, rapamycin can also increase the stability of fully differentiated human Tregs *in vitro* (29), and of adoptively transferred non-human primate Tregs *in vivo* (50). These data provide a strong rationale to consider using rapamycin therapy to promote Treg function *in vivo*.

### The role of cytokines in Treg homeostasis

After development, naïve and memory Tregs in both mice and humans continue to rely heavily on IL-2 signaling for survival and homeostasis. IL-2 may also be important for facilitating Treg survival because it upregulates expression of pro-survival protein myeloid leukemia cell differentiation 1 (MCL1), which counter-regulates the FOXP3-induced pro-apoptotic protein BCL-2-interacting mediator of cell death (BIM) (51, 52). Indeed, administration of IL-2 to mice enhances Treg survival *in vivo* and reduces expression of the pro-apoptotic protein caspase 3 (53). In mice and humans, IL-2 also maintains Treg function by inducing FOXP3 mRNA, stabilizing FOXP3 protein expression, and regulating key Treg-signature molecules such as CTLA-4 and glucocorticoid-induced tumor necrosis factor receptor related protein (GITR) (11).

IL-2 is also essential to prevent the polarization of Tregs into pro-inflammatory effector cells (54, 55). For example, IL-2 signaling in Tregs is required to sustain expression of the GATA-binding protein 3 (GATA3) transcription factor (55, 56). Although this protein is commonly thought of as a Th2 cell lineage-defining protein, its expression is required for negative regulation of the *TBX21* and *RORC* loci, which encode two transcription factors that feedback to diminish FOXP3 expression (55). It is currently not clear whether the role of GATA3 in Tregs is due to direct binding of GATA3 to regulatory regions in the *TBX21* and *RORC* loci, or indirect via positive regulation of FOXP3 itself, which can then repress *TBX21* and *RORC* transcription.

Whether or not other cytokines that signal via γ_c_ can substitute for IL-2 during pTreg development/survival *in vivo* remains unclear. Of note, some murine memory Tregs residing in the skin, or accumulating with age seem to preferentially rely on IL-7 or IL-15 for homeostasis (57, 58). Although human Tregs can definitely proliferate in response to IL-15 (59, 60), the relevance of IL-7 in humans is unclear as the lack of IL7Rα expression is a defining feature of human Tregs (4, 5).

Because of the essential role of exogenous IL-2 for keeping Tregs alive and maintaining FOXP3 expression, therapeutic approaches that deliver IL-2 signals specifically to Tregs are being actively explored. For example, delivery of IL-2/anti-IL-2-antibody complexes in pre-clinical studies stimulates Treg expansion and reduces disease in models of type 1 diabetes (T1D), experimentally induced autoimmune encephalomyelitis (EAE), collagen-induced arthritis, and angiotensin II-induced aortic stiffening (61–64). Similarly, in models of proteinuric kidney disease and renal ischemia-reperfusion injury, administration of IL-2/anti-IL-2-antibody complexes promotes Treg expansion, improves renal function, and reduces inflammation and disease symptoms (65, 66). In clinical trials, low-dose IL-2 therapy has been investigated for the treatment of graft versus host disease (GVHD) and T1D and appears to successfully expand the circulating Treg cell pool (67–70). A major caveat, however, is finding a dose regimen of IL-2 that only affects Tregs and does not activate CD8^+^ T cells and NK cells in parallel, as recently observed in a clinical trial of low dose IL-2 and rapamycin in T1D (71, 72). Another consideration is that IL-2-based therapies might not work in subjects who have genetic defects in IL-2R-signaling such as patients with a T1D-susceptibility IL-2RA haplotype (73) or whose Tregs have become IL-2-unresponsive (74).

A converse application of IL-2 targeted therapy is blockade of IL-2, which could theoretically be beneficial in the setting of cancer where depletion of Tregs could boost anti-tumor immunity (75). Interestingly anti-CD25 mAbs (basiliximab, daclizumab) were originally developed as immunosuppressive agents designed to deplete effector T cells and are still in common use today in transplantation. Investigation into whether daclizumab may also affect Tregs has revealed that it does indeed cause a reduction in Tregs by approximately 50%, both in the setting of autoimmunity (multiple sclerosis) and in cancer immunotherapy (76, 77). Basiliximab has similar effects in transplantation (78). However, post daclizumab therapy, the remaining 50% of Tregs are fully functional (77). These data suggest that, at least using current agents, IL-2 blockade is actually not a very effective way to deplete Tregs, possibly because of their elevated expression of the high affinity IL-2 receptor (i.e. CD25) and/or the ability of other cytokines to compensate *in vivo*.

Another cytokine that has recently gained interest as a regulator of Treg biology is IL-33, a member of the IL-1 cytokine family that signals via a heterodimeric receptor consisting of interleukin-1 receptor-related protein ST2 and the IL-1 receptor accessory protein IL1RAcP (79). Expressed by stromal and immune cells, IL-33 is well known to have a pathological role in airway inflammation and arthritis because it enhances and prolongs immune activation (80). Surprisingly, however, IL-33 treatment can actually protect against experimental colitis and rejection of HLA-mismatched cardiac allografts in mice by promoting Th2 cells and FOXP3^+^ Tregs (81–83). This anti-inflammatory effect of IL-33 on Tregs seems to be mediated in part via DCs, as IL-33-dependent expansion of murine ST2^+^FOXP3^+^ Tregs requires secretion of IL-2 by ST2^+^ DCs exposed to IL-33 (84).

In some tissues, however, there may be direct effects of IL-33 on Tregs. For example, more than 50% of colonic Tregs express ST2 enabling them to quickly respond to IL-33 released by epithelial cells upon tissue damage (85). Functionally, IL-33 can increase TGF-β-induced proliferation of colonic ST2^+^ Tregs *in vitro* and stabilize FOXP3 expression in inflamed tissues *in vivo* (85). Notably, IL-23, which is known to inhibit pTreg differentiation (86, 87), reduces expression of ST2 on Tregs (85), resulting in abrogation of the IL-33-mediated increase in pTreg induction and stabilization. Therefore, the balance between IL-33 and IL-23 may be an important factor in determining the outcome of tissue localized immune responses. In humans, IL-33 was previously thought to be an attractive target for therapeutic blocking (88), as a variety of inflammatory diseases feature elevated serum levels of IL-33. However, in light of its newly discovered function in promoting Treg expansion and function, inhibition of IL-33 could also have deleterious effects in some settings.

### Control of Treg function by cytokines

The function of Tregs is also controlled by local the cytokine milieu, with mounting evidence that the presence of pro-inflammatory cytokines affect Treg suppression both directly and indirectly. Cytokines with direct effects on Tregs, such as tumor necrosis factor alpha (TNF-α), provide possible therapeutic targets for modulating Treg function. TNF-α is a pleiotropic cytokine that can act on a wide range of cells. Tregs express the TNF receptor, and there is evidence for both positive and negative effects of TNF-α on their function. Recent evidence shows that TNF-α induces expression of protein phosphatase 1 (PP1), which de-phosphorylates the C-terminal DNA-binding domain of FOXP3, resulting in a reduction in its function as a transcription factor (89). Notably, treatment of rheumatoid arthritis subjects with TNF-α-antibodies restores Treg function, decreases PP1 expression, and increases FOXP3 phosphorylation. These data are consistent with previous studies showing that TNF-α impairs Treg function in rheumatoid arthritis by reducing FOXP3 expression (90), and that Tregs expressing membrane-bound TNF-α are less suppressive than TNF-α negative Tregs (91). TNF-α also impairs TGF-β-induced pTreg development in EAE by reducing FOXP3 transcription (92).

Data reporting negative effects of TNF-α on Tregs contrast to a series of reports showing that TNF-α signaling through the TNF receptor 2, which is expressed by a subset of mouse and human effector and memory Tregs, enhances Treg proliferation and suppressive activity (93, 94). Notably, one of the common side effects of TNF-α therapy is psoriasis (95, 96) and data from mouse models suggest this may be due to an anti-TNF-α-mediated decrease in Treg frequency in the skin (97). Therefore, environmental TNF-α may actually bolster Treg function. Understanding how the local tissues define whether TNF-α has a negative or positive effect on Treg function will be key to understanding the side effects of this very common therapy.

Similar to conventional CD4^+^ T cells, Tregs respond to lineage-defining cytokines, resulting in differentiation into subsets that seem to mirror classical Th1, Th2, and Th17 cells (98, 99). Th1-like, Th2-like, and Th17-like peripheral CD4^+^CD45RO^+^CD127^low^CD25^high^ memory Tregs can be identified in human peripheral blood on the basis of differential expression of the chemokine receptors CXCR3, CCR4, and CCR6, respectively (100). A major question is whether these subsets of Th-like Tregs are protective or pathogenic. Evidence for the former comes from studies showing that Th-like Tregs remain suppressive and are necessary to provide protection from various diseases (101–114). On the other hand, in humans with autoimmunity and/or inflammation, Th1-like FOXP3^+^ Tregs that express T-bet, CXCR3 and produce IFN-γ appear to lose their suppressive function (115, 116), and multiple reports have shown that Th17-like Tregs are enriched at inflammatory sites, indicating a potential role in disease pathogenesis (116–120). We have also recently shown the first evidence for IL-13^+^ Th2-like Tregs, which are significantly increased in the skin, but not the blood, of subjects with systemic sclerosis (121). IL-13 is a pro-fibrotic cytokine that drives tissue fibrosis in this disease and *in vitro* experiments revealed that IL-33 increases the proportion of IL-13-producing Tregs in cultures of skin biopsies from healthy controls. Therefore, in addition to promoting Treg survival as described above, in some cases, IL-33 may cause detrimental changes to Treg function.

## Metabolites

Dietary metabolites are another important environmental factor that influence Treg differentiation and function, especially in the gut. Research on the effect of metabolites on Tregs has particularly focused on vitamins A, D, and tryptophan. Understanding the effect of these and other metabolites on Tregs could identify dietary supplements that enhance Treg-based therapies and novel compounds that enhance *in vitro* expansion of stable Tregs.

### Vitamin A

All trans retinoic acid (ATRA) is the main bioactive metabolite of vitamin A and, as briefly discussed above, is well known to have an important role in the differentiation of pTregs (122, 123). *In vivo*, a major source of ATRA appears to be mucosal DCs which in mice characteristically express CD103 (integrin α_ε_β_7_), (44–46, 124–126). Since mucosal DCs also express integrin α_v_β_8_, which converts extracellular latent TGF-β to its active form (41), these cells can drive the synergistic induction of FOXP3^+^ pTregs, which specifically express gut homing markers, including CCR9 and integrin α_4_β_7_ (46). In humans, it has recently been demonstrated that ATRA acts on DCs and gives them the ability to preferentially drive the induction of gut homing Tr1 cells, an IL-10-producing FOXP3^−^ Treg subset (127). The Tr1 cells produced by ATRA-producing DCs in this study displayed *in vitro* suppressive function, expressed gut homing markers CCR9 and integrin α_4_β_7_ and also produced IFN-γ.

As described in the Section “Cytokines,” Tregs can convert into Th-like cells in response to different inflammatory cytokines, a phenomenon, which may prove to be an obstacle for their use as immunotherapy. Recent work by Lu et al. has demonstrated that pre-treatment of human CD4^+^CD25^high^CD127^low^ Tregs with ATRA almost completely prevents IL-1β/IL-6-driven conversion to Th1/Th17-like cells (128). Upon *in vitro* expansion in the presence of IL-1β and IL-6, ATRA-primed Tregs maintained high FOPX3 expression, suppressive function and were superior to untreated Tregs in preventing xenogeneic GVHD in mice. A similar effect of ATRA has also been observed in a study of individuals with autoimmune hepatitis type 2. Holder et al. demonstrated that the suppressive function of Tregs specific for liver enzyme cytochrome P450IID6 (the main disease autoantigen) was impaired following culture with IL-1β/IL-6; however, this was prevented by the addition of ATRA (129). Therefore, ATRA appears to be important for Tr1 cell differentiation in the gut and for stabilizing Tregs under inflammatory conditions, and has shown potential for therapeutic use in mouse models of colitis and periodontitis (130, 131).

### Vitamin D

Vitamin D metabolites have long been recognized as important immunomodulators and exert their effects by binding to the vitamin D receptor, which is expressed on many immune cells including activated T cells (132). The active vitamin D metabolite calcitriol (1,25-dihydroxy vitamin D_3_) can be metabolized from vitamin D in the diet or synthesized in the skin following UV exposure. Calcitriol is known to promote the growth of both FOXP3^+^ and IL-10 producing Tregs, while inhibiting Th17 cells (133, 134). It has recently been shown that calcitriol also induces expression of skin (CCR10 and CLA) and inflamed tissue (CXCR6) homing receptors in Tregs (135), and that addition of TGF-β enhances calcitriol-driven expansion of FOXP3^+^ Tregs *in vitro* (136). Furthermore, calcidiol (25-hydroxy vitamin D_3_), a vitamin D metabolite similar to calcitriol, has been shown to prime mucosal DCs to induce suppressive IL-10 and IFN-γ producing Tr1 cells (127). A recent study has suggested that calcitriol could be a useful adjunct therapy with allergens in sublingual immunotherapy as it specifically enhanced Treg responses to allergens *in vitro* (137). It is interesting to note that, following treatment with UVB, MS patients had enhanced levels of serum calcitriol, which correlated with increased levels of circulating pTregs (138). This link between UVB exposure, serum levels of vitamin D metabolites, and Treg frequency might contribute to the observed epidemiological associations between environmental UVB exposure and incidence of autoimmune disease (139–141).

### Metabolites that activate aryl hydrocarbon receptors

Numerous metabolites have been described that can activate the aryl hydrocarbon receptor (AHR), a transcription factor that alters the balance between Tregs and Th17 cells. The direction of this balance shift is though to be ligand-dependent, with some AHR ligands preferentially promoting Tregs and others promoting Th17 cells (142–144). For example, kynurenine, which is produced when tryptophan is catabolized by indoleamine 2,3-dioxygenase (IDO), is an AHR agonist that is important for generating Tregs and inhibiting Th17 cell development (145). Indeed, many tolerogenic cells, such as plasmacytoid DCs (146, 147) produce IDO, and through the production of tryptophan metabolites preferentially induce Tregs. Notably, both IDO and AHR are highly expressed in human placenta, implying that tryptophan metabolites acting via AHR also induces Tregs in pregnancy (148), a process critical for maternal/fetal tolerance (149).

Another tryptophan metabolite, cinnabarinic acid, has been identified as a novel AHR ligand (150) that is also an agonist of the type-4 metabotropic glutamate receptor. Cinnabarinic acid has been shown to prevent onset of EAE in mice following administration of myelin oligodendrocyte glycoprotein peptide through enhancing immune responses that were dominated by Tregs (151). Other dietary metabolites that can act as AHR ligands and promote Tregs include indole-3-carbanole (I3C) and 3,3’-diindolylmethane (DIM), derived from cruciferous vegetables. Treatment of EAE mice with either I3C or DIM completely protects against disease symptoms, significantly reduces immune cell infiltration into the CNS, increases Tregs, and reduces Th17 cells (152). These effects are AHR-dependent as treatment with an AHR antagonist reversed the protective effects of I3C and DIM. Similarly, a study of methionine–choline-deficient (MCD)-diet induced mouse non-alcoholic steatohepatitis (NASH) found that administering DIM reduced disease and shifted the immune dominance from Th17 cells toward Tregs using AHR-dependent mechanisms (153).

Studies of these natural metabolites have also led to the identification of novel AHR ligands, such as benzimidazoisoquinolines, which are not part of a normal diet. Administration of these compounds to mice increased Treg frequency and suppressed GVHD in an AHR-dependent manner (154). Understanding how the activity of AHR controls the balance between Tregs and inflammatory T cells will lead to new approaches to alter this balance therapeutically (155).

### Purine metabolism

Another important metabolic process is purine catabolism, which regulates the balance of pro-inflammatory adenosine 5’-triphosphate (ATP) and immunosuppressive adenosine. Through expression of adenosine receptors and the ecto-enzymes CD39 and CD73 that metabolize ATP, Tregs are able to both react to, and modulate, immune purinergic signals. CD39 and CD73 function to sequentially catabolize extracellular ATP: CD39 catalyzes the conversion of ATP into adenosine diphosphate (ADP) and adenosine monophosphate (AMP); and CD73 converts AMP into adenosine (156). Whereas ATP signals through type 2 purinergic (P2) receptors to initiate pro-inflammatory responses, adenosine, signals through type 1 purinergic (P1) receptors to suppress immune responses (157).

Mouse Tregs express both CD39 and CD73 and the production of adenosine by these enzymes is thought to be one of the Treg mechanisms of suppression (158). In contrast, most human CD39^+^ Tregs do not express CD73, and it is thought that human Tregs primarily generate adenosine when they are in proximity to CD73^+^ cells (159). Interestingly, expression of CD39 enables DCs and neutrophils to move along an ATP concentration gradient to sites of inflammation (160–162), and this may also be true for Tregs. Evidence using the EAE model showing that CD39 has an important role in directing migration of Tregs to lymphoid draining sites of the central nervous system (163) supports this hypothesis.

Regulatory T cells themselves can also respond to adenosine (157), which signals through the A2 class of P1 receptors to stimulate a positive feedback loop by increasing expression of *CD73* mRNA via stimulation of cyclic AMP (cAMP) response elements in the *CD73* locus (164). Adenosine can also act in an autocrine manner via A2A receptors expressed on Tregs to enhance their generation, CTLA-4 expression, and suppressive function (165).

Interestingly, in comparison to the negligible levels of cAMP levels in conventional T cells, human Tregs generate and maintain high intracellular levels of cAMP (166–168). CD39 may be important in this process, as intracellular production of cAMP is increased by extracellular adenosine signaling through A2 receptors. Of note, Tregs can mediate suppression by transferring cAMP through gap junctions into neighboring conventional T cells and DCs (166, 169). Intracellular cAMP also positively feedbacks on Tregs stimulating upregulation of both CTLA-4 (170) and CD39 expression (171).

In terms of the effects of cAMP on pTreg differentiation, in mice there may actually be negative effects as cAMP can suppress TGF-β-driven differentiation of pTregs *in vitro* (172). This negative effect of cAMP is likely due to cAMP-mediated activation of protein kinase A, which enhances TGF-β-mediated activation of mitogen-activated protein kinases ERK and JNK. In humans, however, the effect of cAMP on pTreg differentiation may be different as studies of prostaglandin E2 and vasoactive intestinal peptide, compounds that increase cAMP levels, result in increased pTreg generation and function (172, 173). As growing evidence shows that signaling through G-protein coupled receptors, which stimulate the cAMP pathway, has major effects on Treg differentiation and function (discussed in Section “Microbiome”), developing a full understanding of how cAMP affects Treg biology will be an important area of future research.

## Microbiome

There are approximately 1000 species of different microbes colonizing the gut, with densities of 10^4^-10^5^ bacteria per millimeter of effluent in the proximal small intestine and 10^11^ bacteria per gram of luminal content in the colon (174). The high microbial content in the large intestine poses a large challenge to the mucosal immune system, as it needs to tolerate commensal microbiota and dietary antigens while maintaining the ability to eliminate pathogens. Induction of colonic Tregs is crucial in fostering this immune homeostasis.

**Figure 1:**
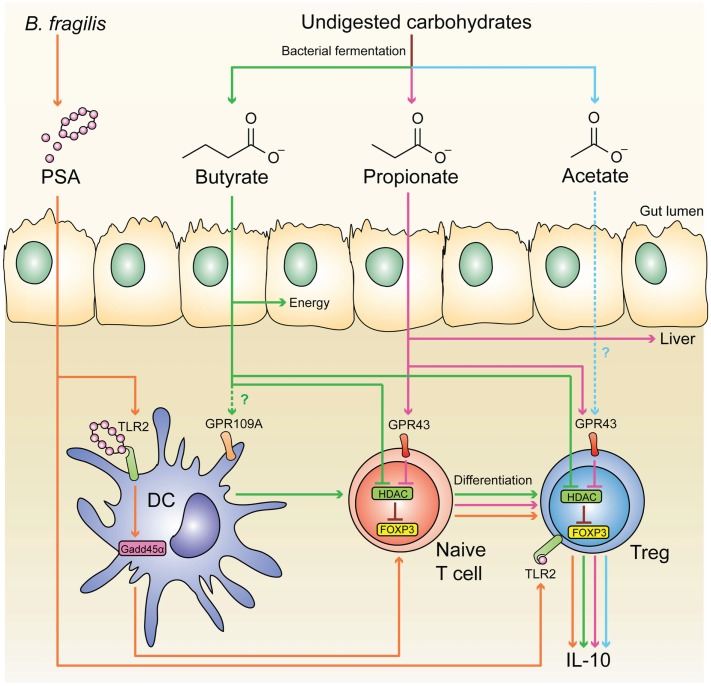
**Microbial-derived molecules promote colonic Treg differentiation**. Undigested dietary carbohydrates are fermented by gut commensal bacteria to produce the SCFAs acetate, propionate, and butyrate. Administration of acetate in drinking water results in the accumulation of IL-10^+^ colonic Tregs, and this effect is independent of HDAC inhibition and acetylation of the *Foxp3* CNS1 region. Although acetate is a potent GPR43 ligand, it is not clear whether acetate mediates its effect through this receptor. GPR43 expression in colonic Tregs is required for propionate to inhibit HDAC function and enhance FOXP3 expression, thereby promoting Treg differentiation and IL-10 production, Butyrate has similar effects by either directly acting on Tregs or through modulating DC function to enhance their Treg-inducing ability; however, the role of GPR109A in these effects is controversial. Purified PSA derived from *B. fragilis* can also directly act on Tregs through TLR2 to promote Treg function by enhancing expression of effector molecules including IL-10, TGF-β2, and granzyme B. Membrane-bound PSA cannot act directly on Tregs, instead it interacts with TLR2 on DCs to promote Treg differentiation in a Gadd45α-dependent manner.

It is now appreciated that a major site for development of pTregs is the colon, resulting in a large population of regulatory cells that have a distinct TCR repertoire and are critical for intestinal homeostasis (175). Since colonic pTregs are significantly reduced in germ-free mice, commensal microbiota has an essential role in inducing these cells (175–177). Similarly, the development of pTregs in the liver (178) and lungs (179) also requires the presence of commensal microbiota early in life. The exact mechanism behind the induction of colonic pTregs remains unknown, but several microbial components have been found to enhance their expansion and function, including short-chain fatty acids (SCFAs) (180–182), and the bacterial molecule polysaccharide A (PSA) of *Bacteroides fragilis* (183, 184) (Figure 1).

### Short-chain fatty acids

Despite substantial individual variation in the composition of the microbial community, in healthy adults most gut bacteria belong to two phyla: Firmicutes and Bacteroidetes (185). Because of the large anaerobic community and low oxygen availability in the colon, bacterial metabolism is dominated by fermentation and anaerobic respiration where nitrate, sulfate, and other compounds are used as electron acceptors (186). Undigested dietary carbohydrates are fermented to produce gases and organic acids, particularly the SCFAs acetate (C2), propionate (C3), and butyrate (C4), typically at a ratio of 3:1:1, respectively (187). Of these three main SCFAs, acetate can be produced by enteric bacteria and acetogens; propionate is a by-product of the succinate pathway in Bacteroidetes; and butyrate is formed from two acetyl-CoA molecules in Firmicutes (188). Specific species that have been recognized by their high levels of butyrate production include *Faecalibacterium prausnitzii* and the cluster IV and XIVa of genus *Clostridium* (189). SCFAs are the most abundant (50–100 mM) in the proximal colon, where most fermentation occurs (190). However, in the peripheral blood, only acetate remains in relatively high concentrations, since butyrate is preferentially utilized by colonocytes as an energy source and propionate is metabolized by hepatocytes (190).

Recent work has revealed that SCFAs are important in promoting the differentiation of colonic Tregs. An early study reported that germ-free mice have reduced colonic Treg numbers, and that colonization by bacterial strains belonging to the cluster IV and XIVa of the genus *Clostridium* rescues the deficiency and protects mice from colitis (176). Administration of acetate, propionate, and butyrate in drinking water mimics the effect of *Clostridium* colonization in germ-free mice, resulting in an elevated Treg frequency in the colonic lamina propria and increased IL-10 production by these Tregs (180, 182).

Of the three main SCFAs, butyrate has been found to be the most potent inducer of colonic Tregs. Mice fed a diet enriched in butyrylated starches have more colonic Tregs than those fed a diet containing propinylated or acetylated starches (181). Arpaia et al. tested an array of SCFAs purified from commensal bacteria and confirmed butyrate was the strongest SCFA-inducer of Tregs *in vitro* (180). Mechanistically, it has been proposed that butyrate, and possibly propionate, promote Tregs through inhibiting histone deacetylase (HDAC), causing increased acetylation of histone H3 in the *Foxp3* CNS1 region, and thereby enhancing FOXP3 expression (180, 181).

Short-chain fatty acids partially mediate their effects through G-protein coupled receptors (GPR), including GPR41, GPR43, and GPR109A. GPR41 and GPR43 are stimulated by all three major SCFAs (191), whereas GPR109A only interacts with butyrate (192). In mice, colonic and small intestinal Tregs express GPR43, and expression of this receptor is required for propionate-mediated HDAC inhibition and Treg expansion (182). There are conflicting results as to whether GPR109A is required for butyrate to mediate its pro-Treg effect. In both mice and humans, GPR109A is only expressed on DCs and macrophages, but not on T cells (192). Singh et al. found that splenic DCs from *Gpr109a*^−/−^ mice were not able to induce Treg differentiation in response to butyrate (192). Additionally, the study found that butyrate treatment increases transcription of *Aldha1* (aldehyde dehydrogenase) in a GPR109A-dependent manner (192). As discussed above, this enzyme is important in vitamin A metabolism, so these data suggest that GPR109A signaling may increase ATRA production by APCs and indirectly promote Treg differentiation and function. In contrast, Arpaia et al. reported that butyrate-pre-treated *Gpr109a*^−/−^ DCs are not defective in *in vitro* generation of Tregs (180). The reason for these discrepant findings is not clear, but overall the emerging data demonstrating that SCFAs can have both direct and indirect effects on Tregs and have opened up an exciting new area of research.

### Polysaccharide A

Another microbial component capable of enhancing Treg function is PSA from the commensal gut bacterial strain *B. fragilis*. The initial study reported that in germ-free mice either colonization by *B. fragilis* or administration of purified PSA induces IL-10 secretion by CD4^+^ T cells and reduces gut inflammation (193). A subsequent study confirmed that the IL-10-expressing CD4^+^ T cells were FOXP3^+^ Tregs and that PSA treatment increases Treg frequency and their expression of effector molecules including IL-10, TGF-β2, granzyme B, and CCR6 (184). Notably, the authors found that in the absence of APCs, PSA acts directly on Tregs through toll-like receptor 2 (TLR2) to induce the observed effects (194). It has also been demonstrated that administration of PSA protects against induction of EAE in mice through TLR2-mediated expansion of CD39^+^ Tregs (195).

It remains unknown how PSA is recognized by the mucosal immune system. Since the genome of *B. fragilis* does not contain genes for any known bacterial secretion system (196) and PSA is a large capsular polysaccharide (197), it has been proposed that *B. fragilis* delivers PSA by secreting outer membrane vesicles (OMVs) (198). Shen et al. observed that oral administration of PSA-containing OMVs purified from *B. fragilis* is sufficient to protect mice from experimental colitis and that TLR2 expression on DCs, but not T cells, is required to promote IL-10 production by Tregs (198). Subsequent work identified that PSA-treated plasmacytoid DCs, but not conventional DCs, are responsible for inducing IL-10-secreting Tregs (199). Therefore, while Tregs can directly respond to purified PSA, immune responses to membrane-bound PSA require TLR2^+^ DCs. It is worth noting that many other TLR ligands can also directly or indirectly impact Treg function [reviewed in Ref. (200)]. In contrast to PSA, other TLR2 ligands, such as Pam3CSK4 and FSL-1, inhibit the function of both mouse and human Tregs (201–204). How multiple TLR signals are integrated in the mucosal environment is unknown.

### Probiotics

Long before the direct effects of microbial products on Tregs were understood at the molecular levels, many groups have been exploring the potential therapeutic use of bacteria in the form of probiotics to modulate Tregs. For example, administration of a five-strain probiotic mixture (designated IRT5, including *Lactobacillus acidophilus*, *Lactobacillus casei*, *Lactobacillus reuteri*, *Bifidobacterium bifidum*, and *Streptococcus thermophilus*) in mice increases the proportion of Tregs in the mesenteric lymph nodes (205). CD11c^+^ DCs purified from these treated mice also had higher expression of IL-10, TGF-β, and IDO, and were more capable of inducing Treg differentiation compared to DCs from control mice (205). A more recent study demonstrated that administration of *L. reuteri* to mice was sufficient to prevent high-fat-diet-induced adipose inflammation and obesity, an effect that was associated with enhanced Treg induction and IL-10 expression (206). *In vitro*, *L. casei* and *L. reuteri* can also prime human monocyte-derived DCs to stimulate IL-10-producing Tregs through the adhesion molecule DC-SIGN (207). Lopez et al. found that DCs exposed to *B. bifidum* membrane vesicles strongly induced Treg differentiation *in vitro*, suggesting that the potential use of the membrane vesicle as a safe adjunct therapy (208).

Although much work is still needed to elucidate the details of how commensal microbiota induce Tregs, numerous randomized trials in the past decade using *Lactobacillus* and *Bifidobacterium* to treat inflammatory disorders have already demonstrated the clinical benefit of this approach (209). Indeed, while delivery of purified PSA or SCFAs may represent an effective, transient therapy, the use of probiotics may offer a well-tolerated long-term therapeutic solution to enhancing intestinal immunoregulatory cells.

## Conclusion

Environmental stimuli influence all aspects of Treg biology: from development and differentiation to migration and function. As well as refining our understanding of how well-described cytokines affect Tregs, we are also discovering new cytokines, such as IL-33, which have a critical role in Treg function (Table 1). Other key factors influencing Tregs, particularly in the gut, are dietary metabolites, catabolites, and bacterial components from the microbiome. There is emerging evidence that retinoic acid is a key metabolite for expanding a stable population of Tregs, data that have clear implications for developing therapeutic approaches. Furthermore, aspects of the microbiome clearly help determine which commensal antigens the immune system is educated against and have a previously unappreciated role in influencing T cell differentiation in the gut. Further studies in this area will expand our knowledge of T cell biology and hopefully uncover new elements of disease pathogenesis and guide the development of Treg-based therapies.

## Conflict of Interest Statement

The authors declare that the research was conducted in the absence of any commercial or financial relationships that could be construed as a potential conflict of interest.
